# Administration of Reconstituted Polyphenol Oil Bodies Efficiently Suppresses Dendritic Cell Inflammatory Pathways and Acute Intestinal Inflammation

**DOI:** 10.1371/journal.pone.0088898

**Published:** 2014-02-18

**Authors:** Elisabetta Cavalcanti, Elisa Vadrucci, Francesca Romana Delvecchio, Francesco Addabbo, Simona Bettini, Rachel Liou, Vladia Monsurrò, Alex Yee-Chen Huang, Theresa Torres Pizarro, Angelo Santino, Marcello Chieppa

**Affiliations:** 1 Laboratory of Experimental Immunopathology, IRCCS “de Bellis,” Castellana Grotte (BA), Italy; 2 Laboratory of Mucosal Immunology, Mario Negri South, Santa Maria Imbaro (CH), Italy; 3 Laboratory of Translational Medicine, ARCHES, Castellana Grotte (BA), Italy; 4 Department of Engineering for Innovation, University of Salento, Lecce, Italy; 5 Department of Pediatrics, Case Western Reserve University School of Medicine, Cleveland, Ohio, United States of America; 6 Department of Pathology and Diagnostic, University of Verona Medical School, Verona, Italy; 7 Department of Pathology, Case Western Reserve University School of Medicine, Cleveland, Ohio, United States of America; 8 Institute of Science of Food Production, CNR, Lecce, Italy; University Heart Center Freiburg, Germany

## Abstract

Polyphenols are natural compounds capable of interfering with the inflammatory pathways of several *in vitro* model systems. In this study, we developed a stable and effective strategy to administer polyphenols to treat *in vivo* models of acute intestinal inflammation. The *in vitro* suppressive properties of several polyphenols were first tested and compared for dendritic cells (DCs) production of inflammatory cytokines. A combination of the polyphenols, quercetin and piperine, were then encapsulated into reconstituted oil bodies (OBs) in order to increase their stability. Our results showed that administration of low dose reconstituted polyphenol OBs inhibited LPS-mediated inflammatory cytokine secretion, including IL-6, IL-23, and IL-12, while increasing IL-10 and IL-1Rα production. Mice treated with the polyphenol-containing reconstituted OBs (ROBs) were partially protected from dextran sodium sulfate (DSS)-induced colitis and associated weight loss, while mortality and inflammatory scores revealed an overall anti-inflammatory effect that was likely mediated by impaired DC immune responses. Our study indicates that the administration of reconstituted quercetin and piperine-containing OBs may represent an effective and potent anti-inflammatory strategy to treat acute intestinal inflammation.

## Introduction

Intestinal inflammatory disorders, including inflammatory bowel diseases (IBD)s, are common multifactorial clinical pathologies resulting from uncontrolled and deregulated gut mucosal immune responses[1]. In fact, the intestinal immune system is characterized by unique features that are specific for surveying the largest exposed surface of the body that is in direct contact with the external environment, and for balancing physiological inflammation while maintaining normal gut homeostasis[2]. Several different cells serve important roles in this complex immune surveillance. Dendritic cells (DCs), for example, play a pivotal role in switching the adaptive immune response from a tolerogenic to an inflammatory state[3]. It has been postulated that triggering oral tolerance in patients with IBD is due, in part, to defective inflammatory cytokine production by intestinal resident DCs[4].

Intestinal DC cytokine production is unique and imprinted by exposure to epithelial factors, including TSLP and TGFβ[5] [6] [7]. Disruption of the epithelial barrier changes the gut milieu towards an inflammatory phenotype that facilitates intestinal DC accumulation to the site of inflammation[8] [9] [10] and promotes the secretion of inflammatory mediators, including TNFα, IL-12, and IL-18[11] [12]. Taken together, various therapeutic approaches that target inflammatory DCs by dampening proinflammatory cytokine production have been evaluated[13]. Clinical therapies currently available include, for example, the systemic administration of antibodies against TNFα. Treatment with anti-TNF, in fact, is proven to be effective even in cases of chronic disease; however, a significant percentage of patients do not respond or become resistant to anti-TNF therapy[14].

Several studies have described the beneficial effects of plant-derived polyphenols as natural ligands that are able to reduce inflammation, with some inhibiting production of TNFα from cell lines of different origins in both *in vitro* and *in vivo* models[15]. Little is known, however, regarding the immunomodulatory effects of polyphenols on DCs, likely due to their chemical characteristics that impart instability, particularly in an *in vivo* setting, and limit their translational potential[16] [17]. Quercetin, similarly to many other phytochemicals, is a hydrophobic compound characterized by low solubility in water and consequent low bioavailability. These major limitations can be bypassed by developing efficient delivery systems that have the ability to protect, as well as release, polyphenols at the appropriate site of action. A wide variety of new delivery systems has been proposed, including liposomes, nanoparticles, and nanoemulsions[18]. Among these, plant OBs represent a convenient and feasible option to achieve the aforementioned goals. OBs are lipid storage vesicles that are naturally found in plant seeds. Isolated OBs are remarkably stable due to the steric hindrance and electro-negative repulsion provided by surface proteins of the organelles[19] [20]. According to the relative proportions of triacylglycerols (TAGs), PLs, and proteins, stable OBs can be technically reconstituted with these three essential constituents[21]. Reconstituted oil bodies (ROBs), as well as native OBs, have been previously reported as useful vehicles for the stabilization of curcumin, another polyphenol[22].

In the present study, we pre-selected the most effective combination of polyphenols to suppress *in vitro* LPS-induced inflammatory cytokine secretion from DCs. We then encapsulated the selected polyphenols into ROBs that efficiently decreased polyphenol degradation. The combined administration of ROBs containing quercetin and piperine (ROBs-QP) improved the efficacy of suppression of LPS-induced inflammatory cytokine production from DCs, even at a low dose. The two ROBs efficiently synergized to induce significant TNFα suppression. Inhibition of DC inflammatory pathways was confirmed by a decrease in phospho-p38 and COX-2 that was particularly evident when the two nanocapsules were administered together. Finally, we addressed the *in vivo* efficiency by evaluating the ability to protect C57Bl/6 mice from acute colitis induced by 2% DSS administration [23]. In this animal model, administration of ROBs-QP was able to delay weight loss, reduce the inflammatory score, and improve survival. These findings suggest that administration of ROBs-QP suppresses the DC inflammatory program *in vitro* and *in vivo*, thus ameliorating acute colitis and possibly other chronic intestinal inflammatory disorders, and provide the rationale for further *in vivo* usage of the proposed compounds.

## Results

### Quercetin administration during DC maturation inhibits LPS-mediated TNFα and IL-6 production

**Figure 1:**
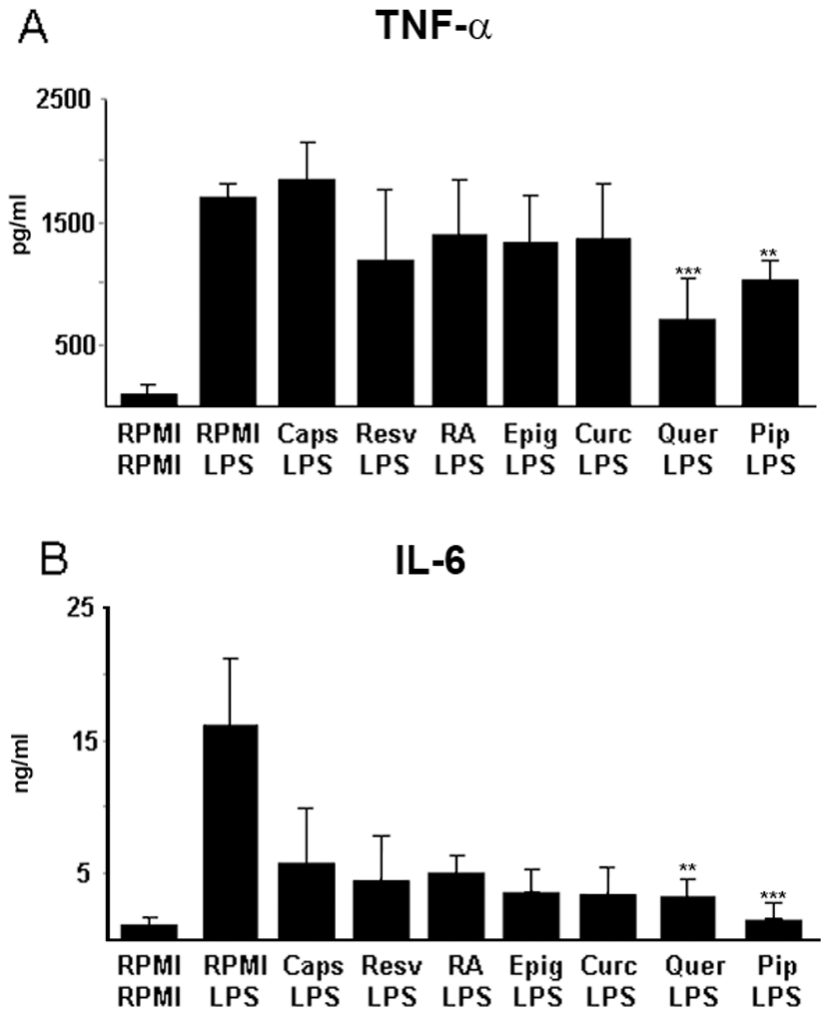
Polyphenol administration to BMDCs interferes with LPS-mediated TNFα and IL-6 production. 25 µM of each polyphenol was administered on day 5 and 7; LPS [1 µg/ml] was administered on day 8. SNs were collected 24 h later, and TNFα and IL-6 protein levels were assessed by ELISA. Both cytokine concentrations were significantly inhibited when cells were exposed to Quercetin (Querc) and Piperine (Pip) when compared to control (RPMI+LPS). Data are shown as mean ± S.D. of five independent experiments. Differences where considered between control (RPMI-LPS) and LPS-Quer- or LPS-Pip-treated cells **P<0.01, ***P<0.001. Capsaicin (Caps), Resveratrol (Resv), Rosmarinic Acid (RA), Epigallocatechin (Epig) and Curcumin (Curc).

We first compared a battery of synthetic and commercially available polyphenols for their ability to inhibit LPS-induced inflammatory cytokine production from DCs. Bone marrow-derived DCs (BMDCs) received 25 µM of a single polyphenol on the third and fifth day of *in vitro* culture. On day 7, DCs were exposed to LPS [1 µg/ml], and 24 hours later, supernatants (SNs) were collected, and IL-6 and TNFα protein levels were determined by ELISA (Fig. 1). Quercetin was consistently the most efficient inhibitor of both TNFα and IL-6 protein production compared to other polyphenols, while piperine appeared to have specificity for IL-6 inhibition. Surprisingly, *trans*-resveratrol, one of the most studied polyphenols, proved to be one of the least efficient inhibitors (Fig. 1).

### ROB encapsulation efficiently stabilizes quercetin

**Figure 2:**
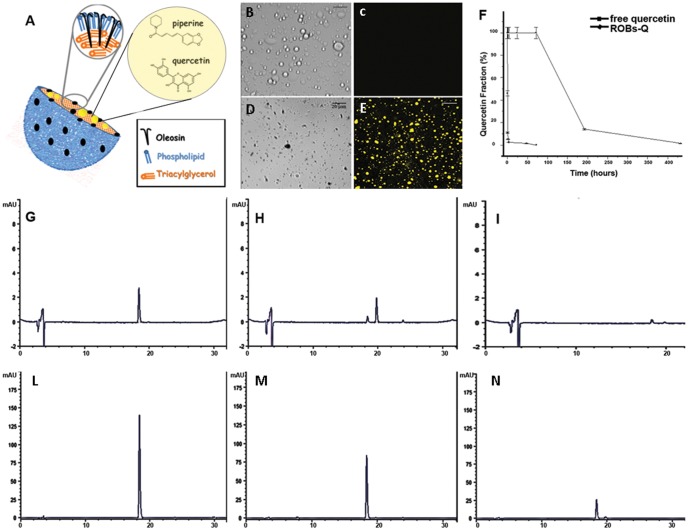
Characterization of polyphenol-embedded ROBs. A) model structure of a natural oil body: external PL monolayer, embedded proteins (*i.e*. oleosin) and TAGs; Piperine and quercetin were encapsulated in the core. B and D) CLSM micrograph of empty ROBs and quercetin encapsulated ROBs imaged by transmitted light. C and E) LSM micrograph of empty ROBs and ROBs quercetin imaged by fluorescence light using an excitation wavelength of 488 nm and emission recorded with a 505–530 nm filter set. F) Degradation profiles of free (squares) and NE (circles) quercetin incubated in 0.1 M phosphate buffer pH 7 at 37°C in the dark for different times; HPLC chromatograms of free quercetin (12.5 µM) incubated with 0.1 M phosphate buffer pH 7 in the dark at 37°C after 0 h (G), 4 h (H) and 48 h (I) compared to HPLC chromatograms of quercetin encapsulated ROBs (12.5 µM) treated in the same experimental conditions at 48 h (2 days, L), 144 h (6 days, M) and 432 h (18 days, N).

Plant OBs share a common structure comprised of a hydrophobic core of triacylglycerols (TAGs) and sterols surrounded by a monolayer of polar phospholipids (PLs) embedded with associated proteins (Fig. 2a). Figure 2F clearly shows rapid polyphenol degradation once dissolved in 0.1 M phosphate buffer of pH 7 at 37°C to a final concentration of 12.5 µM. Free quercetin was almost completely degraded in 4 hours, with approximately 50% degradation at 30 min (Fig. 2F). Furthermore, 4 hours following quercetin incubation in phosphate buffer, the presence of an additional peak, eluting with a retention time of around 20 min (Fig. 2G, H, I), was observed by chromatograms of RP-HPLC analysis. After 48 hours in phosphate buffer, both of these compounds were almost completely degraded (Fig. 2I). These data were further supported by the formation of degradation product upon analysis of UV-Vis absorption spectra obtained at different time points (data not shown). This analysis showed a gradual blue shift from the maximum absorbance at around 370 nm to about 330 nm, confirming the loss of conjugation of the chromophore. These results were in agreement with those already reported by others[24].

Quercetin was then encapsulated into ROBs (ROBs-Q) to improve its stability and bioavailability. ROBs-Q were imaged by confocal laser scanning microscopy (CLSM) and images showed the presence of micro/nanocapsules of expected size and shape (Fig. 2E). Quercetin encapsulated into ROBs showed 100% stability up to three days. After six (144 h) and 10 days (240 h), 50% and 20%, respectively, of the initial active compound were detected (Fig. 2F and L–N). Furthermore, the RP-HPLC elution profiles showed that for ROBs-Q, the degradation product was always a minor peak up to 20 days of incubation (data not shown).

### Polyphenol embedded ROBs potently inhibit inflammatory cytokine secretion from DCs

**Figure 3:**
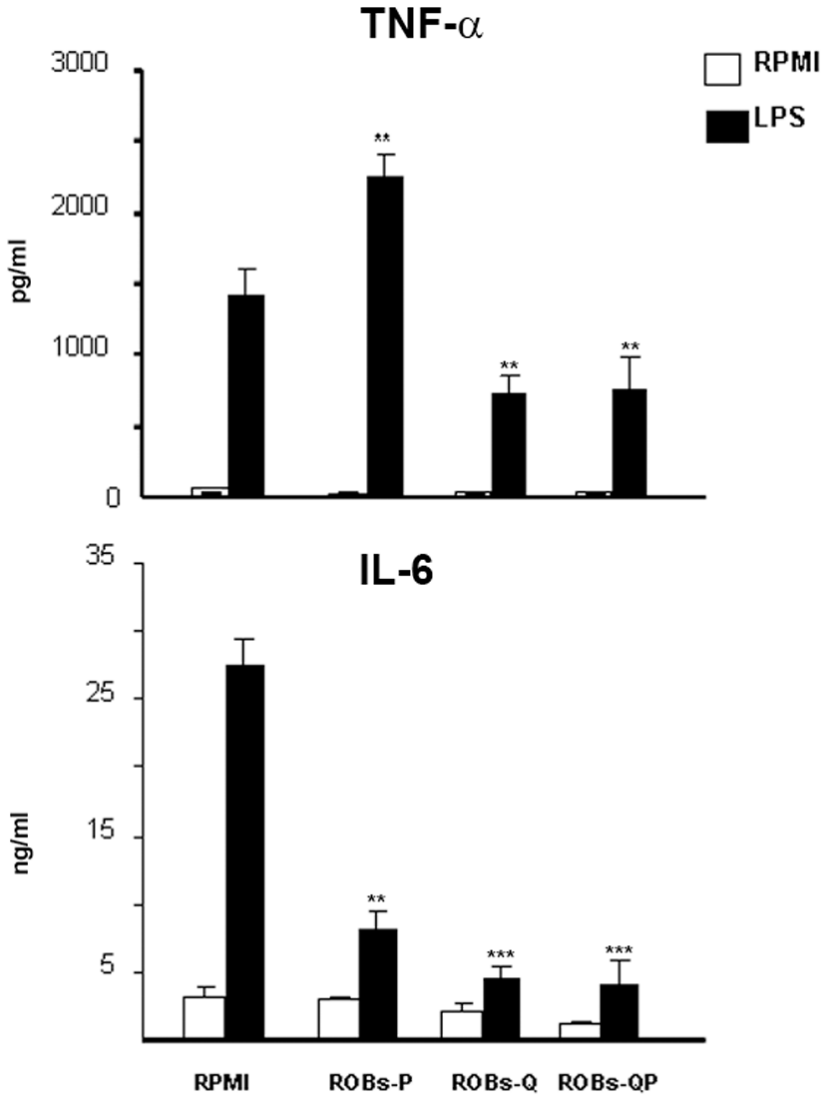
ROB-embedded polyphenol mix interferes with production of LPS-mediated cytokines from DCs. ROB-embedded piperine (ROBs-P), quercetin (ROBs-Q) or a mix of the two (ROBs-QP) were administered on day 5 and 7 [25 µM] to DC cultures. LPS [1 µg/ml] was administered on day 8. SNs were collected 24 h later and TNFα and IL-6 content evaluated by ELISA. Data are shown as mean ± S.D. of five independent experiments; **P<0.01, ***P<0.001.

BMDCs were exposed to several ROBs-QP concentrations to address potential toxicity of the treatment. We observed a significant increase in apoptosis when DCs were treated with 100 µM of ROBs-QP. The percentage of apoptotic DCs obtained from 25 µM ROBs-QP treated wells was always below 18% and never significantly higher than the percentage of apoptotic cells observed in the untreated wells (data not shown). BMDCs were exposed to 25 µM of ROBs-embedded quercetin or piperine (ROBs-Q or ROBs-P, respectively), or ROBs containing the combination of the two polyphenols (ROBs-QP) at the same concentrations. Our results showed that ROBs-QP efficiently inhibited TNFα and IL-6 production (Fig. 3). Of note, ROBs-QP did not affect production of cytokines since cytokine production was comparable to untreated DCs in the absence of TLR4 engagement by LPS. Empty ROBs failed to decrease TNFα and IL-6 protein levels, revealing that the polyphenol content, and not the protective shell, was responsible for inhibition of inflammatory cytokine production (Fig. 3). To exclude eventual induction of cells proliferation, we collected and counted the cells following the supernatant collection. DCs proliferation was never observed (data not shown).

### LPS- and PG-associated ph-p38 is decreased following ROBs-QP administration

**Figure 4:**
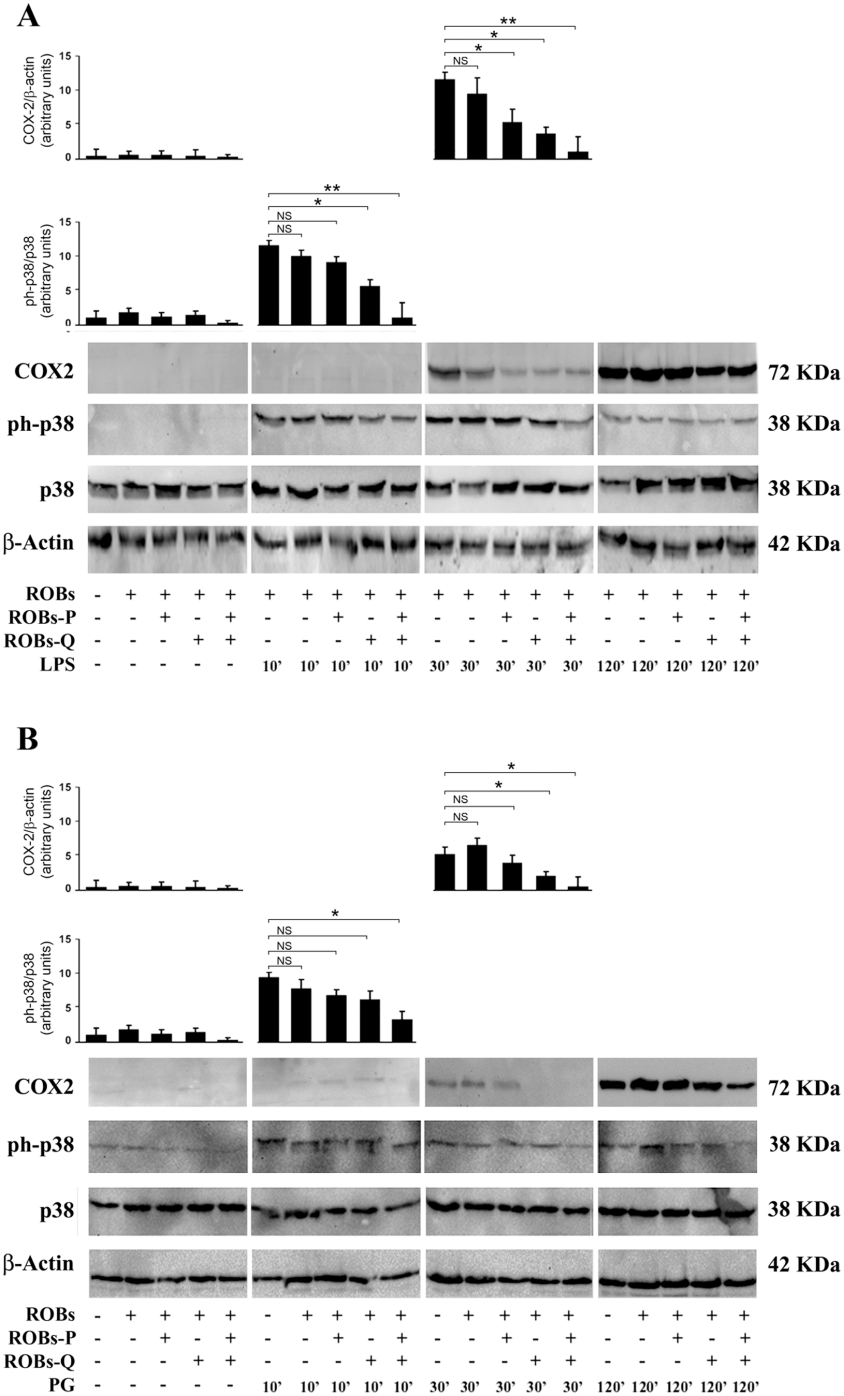
Modulated activation of pro-inflammatory MAPK p38 signaling and inducible COX-2 on ROBs-QP treated DCs. DCs were cultured in the presence of either ROBs-P, ROBs-Q or ROBs-QP as mentioned previously; empty ROBs were used as control. LPS (A) or PG (B) was administered at indicated time points. DC lysates were subjected to immunoblotting with antibodies to detect total and phosphorylated forms of p38 MAPK and COX-2. Representative immunoblots from at least three independent experiments are shown for each condition. Each bar represents the mean ± SEM of densitometric analyses for phosphorylated proteins normalized to their respective total forms; *P<0.05, **P<0.01 vs. basal conditions.

As shown in Figure 4A and B, BMDCs exposed to either LPS or PG acutely activated the MAPK p38 signaling pathway. Treated cells significantly increased levels of p38 MAPK phosphorylation as compared to basal levels. Time course experiments (Baseline, 10′, 30′, 120′) indicated both TLR4- and TLR2-dependent activation of p38 MAPK peaks during the first 10 min and remained sustained for 2 h. Quantitative analysis of phosphorylation levels also revealed a different order of magnitude for LPS- and PG-mediated phosphorylation of p38 MAPK (Fig. 4A and B).

### Administration of low dose ROBs-QP promotes a unique pattern of cytokine production

**Figure 5:**
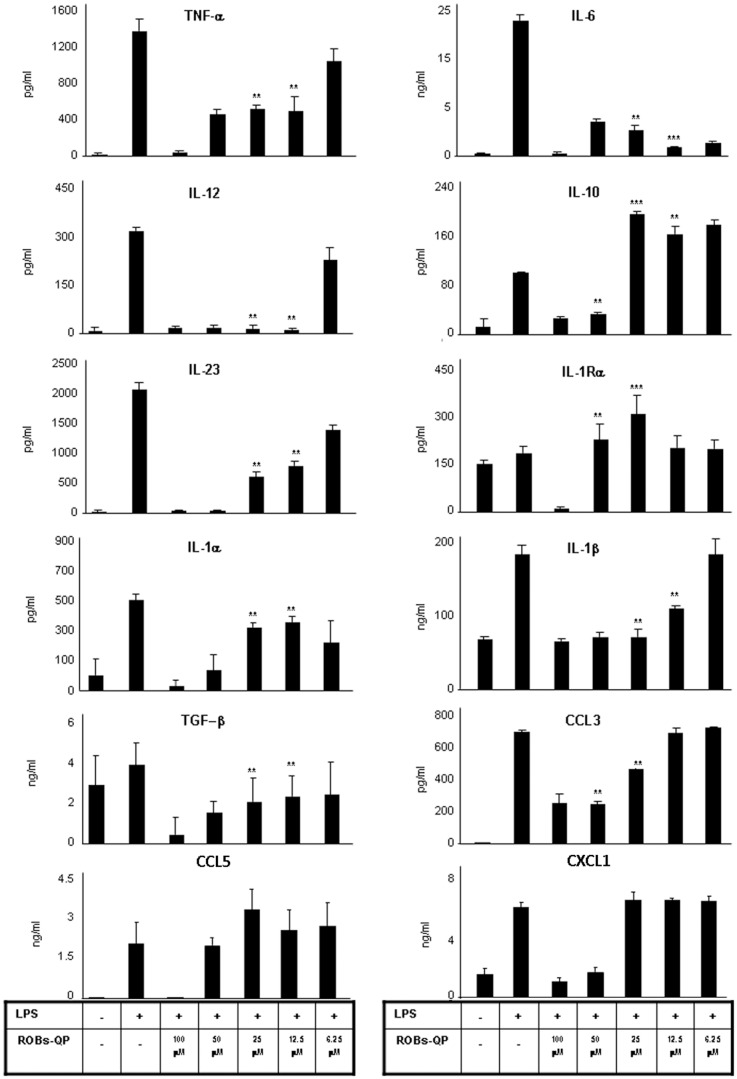
ROBs-QP administration promotes a unique cytokine profile in LPS-treated DCs. BMDCs were exposed to different ROBs-QP concentrations on day 5 and 7. LPS [1 µg/ml] was administered on day 8, and 24 h later SNs were collected. Cytokine protein levels were measured by ELISA. Data are shown as mean ± S.D. of five independent experiments. Statistically significant differences were considered when **P<0.01; ***P<0.001 between control (LPS, no polyphenols) and LPS-ROBS-QP-treated cells.

Most of the *in vivo* studies following polyphenol administration failed translation into clinical practice due to rapid polyphenol degradation in water and the high dosage required to obtain significant results. DCs were treated as previously described using different doses of ROBs–QP, and production of several DC-derived cytokines was evaluated. Figure 5 shows that ROBs-QP effectively inhibits the production of acute phase inflammatory cytokines, including TNFα, IL-6, IL-23 and IL-12. Administration of 100 µM of ROBs-QP reduced DC activity, as demonstrated by decreased cytokine production, and 50 µM ROBs-QP revealed low toxicity as viability was comparable among treated and untreated DCs. Administration of 50 µM ROBs-QP decreased TNFα, IL-12 IL-23, IL-6, CCL3, CXCL1 and even IL-10 production. IL-1Rα, IL-1β, CCL5 and TGFβ secretion was not affected. Administration of 25–12.5 µM ROBs-QP still had the ability to inhibit the acute inflammatory pathway and to induce increased levels of anti-inflammatory cytokines. Efficacy of ROBs-QP was lost at 6.25 µM, with the exception of IL-6 production that was still potently inhibited.

### Intraperitoneal delivery of ROBs-QP ameliorates acute intestinal inflammation induced by administration of 2% DSS

**Figure 6:**
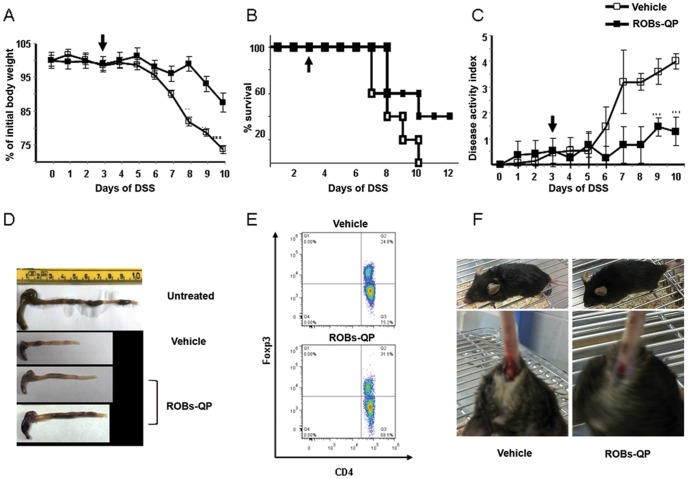
Intraperitoneal administration of ROBs ameliorates 2% DSS-mediated acute colitis. Sex- and weight-matched mice were divided in 2 groups (10 mice each). Mice received 2% DSS in drinking water from day 0, and either ROBs-QP or vehicle starting from day 3. Body weight and presence of diarrhea and rectal bleeding were monitored daily. (A) Average body weight is shown as mean ± SEM. Arrow indicates the beginning of ROBs-QP administration; *P<0.05, **P<0.01. (B) Survival rate is shown as percentage of initial number of either untreated or ROBs-QP-treated mice; N = 10/group, P≤0.01 vs. all treated groups. (C) Disease Activity Index (DAI) was calculated daily. The values represent the mean of 6 mice (3 females and 3 males) ± SEM; *P<0.05, **P<0.01. (D) Macroscopic changes at day 9 of representative colons isolated from mice treated with 2% DSS. (E) At day 9, mice were sacrificed and MLNs explanted, and percentages of CD4^+^Foxp3^+^ cells were assessed by FACS; figure shows a representative dot plot. (F) Fur and perianal beading of representative untreated vs. ROBs-QP-treated mice 9 days after the beginning of 2% DSS administration.

**Figure 7:**
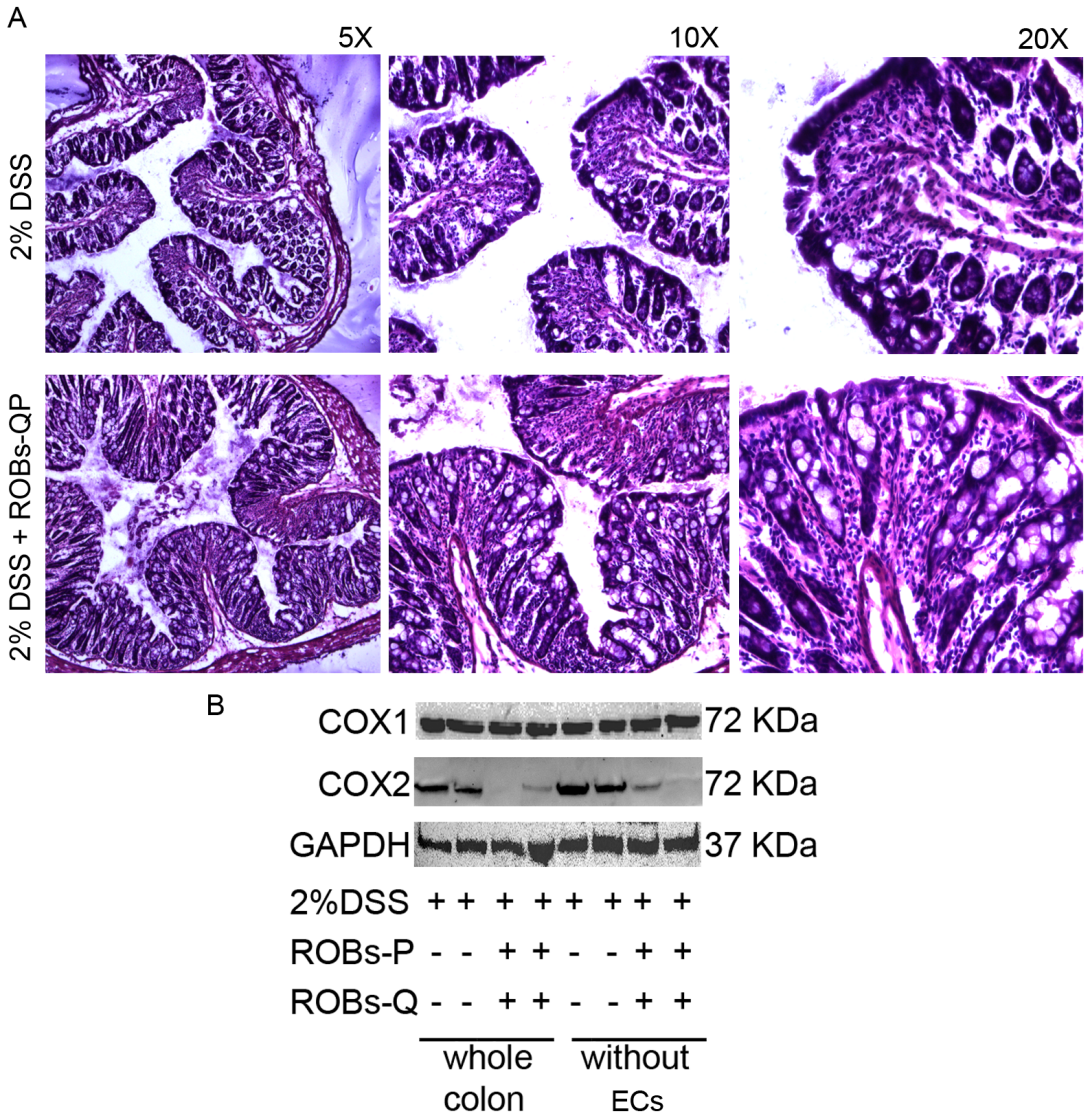
ROBs-QP *in vivo* administration reduced expression of COX2 in colon lysates even in absence of the epithelial cells layer. (A) Histology from ROBs-QP treated mice indicates signs of inflammation and infiltrating granulocytes in the colon of vehicle- and ROBs-QP-treated mice. (B) Western blot staining of colon lysates obtained from mice treated as previously described. Reduced expression of COX2 can be observed with or without epithelial cells from colon lysates of ROBs-QP-treated colons. The constitutive isoform COX1 showed no detectable differences.

The efficiency of polyphenol stabilization following encapsulation of ROBs, combined with an effective dose needed to obtain suppression of acute inflammatory cytokine production, allowed us to design appropriate *in vivo* treatment strategies. Two groups of C57Bl/6 mice received 2% DSS in their drinking water to induce acute colitis[23]. Mice were carefully observed and weight, general health conditions, and fecal blood were monitored daily. Starting from day 3 of DSS colitis, the mice received ROBs-QP by intraperitoneal injection [0.5 µM/g] three times per week. Administration of ROBs-QP effectively delayed the presence of fecal blood and prevented weight loss. In vehicle treated mice, diarrhea and weight loss were detected at day 5, followed shortly after by the presence of fecal blood. In these mice, weight loss at day 6 and day 8 was 5% and 18%, respectively. Administration of ROBs-QP significantly delayed weight loss that was 2% at day 6 and 8, and reached 13% at day 10 (Fig. 6A). Mice in which disease reached maximal severity, defined by the ethical approval protocol for animal research (see methods), were euthanized. ROBs-QP treatment prolonged mice survival (Fig. 6B) and reduced the overall disease activity index (Fig. 6C). At day 9, equal numbers of mice per group were sacrificed, and colon tissues and MLNs were harvested. Colons from ROBs-QP treated mice appeared less inflamed as shown by quantitative parameters, including colonic length and weight (Fig. 6D). From these mice, we evaluated MLNs for the number of CD4^+^Foxp3^+^ cells. ROBs-QP treatment induced a 15% increase in the CD4^+^Foxp3^+^ population. In addition, reduced colon length, increased diarrhea and rectal bleeding occurred in DSS-treated mice; however, all of these symptoms were reduced by ROBs-QP administration. Profound differences between vehicle- and ROBs-QP-treated mice were observed at day 9, and revealed that vehicle-treated mice showed furry hair, while ROBs-QP-treated mice showed normal hair. In addition, the severity of fecal bleeding was vastly reduced in ROBs-QP-treated mice (Fig. 6F). Representative histology obtained from the colon of vehicle- and ROBs-QP-treated mice was observed at day 9 (Fig. 7A). Signs of inflammation and infiltrating granulocytes can be detected in both samples. It is possible to observe a lower grade of inflammation in the samples obtained from ROBs-QP treated mice. We further analyzed COX2 in colon lysates from these same donors. Figure 7B shows reduced expression of the inflammatory mediator COX2 in colon lysates. Consistently with other studies levels of COX-2 was remarkably increased in colon obtained from 2%DSS treated mice. ROBs-QP administration remarkably reduced COX-2 induction, likely via the reduced P38 activation. The constitutive isoform COX1 showed no detectable differences between vehicle- and ROBs-QP-treated colons. Furthermore, we mechanically stripped the epithelial cells layer to investigate the ROBs-QP mediated COX2 reduction in the non-epithelial compartment. As shown in figure 7B, COX2 reduction was still detected in ROBs-QP-treated cells, thus suggesting that the non-epithelial colon compartment is also affected by ROBs-QP administration.

## Discussion

In this study, we evaluated an effective method to obtain alternatively activated DCs by administration of a polyphenol mix, embedded in ROBs. DCs are critical players of the inflammatory response, with the ability to capture, process, and present antigens to direct the adaptive immune response. Upon TLR engagement, immature DCs profoundly change and become mature cells[25] [26]. These cells are commonly characterized by low endocytosis, altered surface marker expression (*e.g*., costimulatory proteins, such as CD80 and CD86), switching of chemokine receptor expression, and cytokine production[27] [28]. As previously discussed, alternatively activated DCs, even upon TLR engagement, do not promote inflammation and are involved in promoting tolerance[29]. In the intestine, a single monolayer of epithelial cells separates billions of bacteria from the largest concentrated population of immune cells [2]. Normal intestinal homeostasis is preserved through dynamic mechanisms[8] [9] [10]. The intestinal milieu is able to educate resident DCs to develop into alternatively activated DCs that will not produce inflammatory mediators, even following TLR engagement[30] [5] [6] [7]. Chronic intestinal inflammation is often characterized by loss of alternatively activated DCs and the presence of pro-inflammatory DCs. The present study was undertaken to explore the possibility of promoting alternatively activated DCs by administration of nutritionally-derived compounds, commonly found in the diet. Several studies have reported that polyphenol intake is associated with reduced risks of cardiovascular and neurodegenerative diseases, as well as cancer development [31] [32] [33] [34]. This action appears to be related to the modulation of NF-kB and MAPK activation[35] [36]. Numerous studies have evaluated the possibility of polyphenols to serve as a potential therapy for patients with IBD [37]. Results from these studies were somewhat inconsistent, and required a high polyphenol concentration in order to obtain significant results *in vivo*.

We first evaluated the effects of different polyphenols using an *in vitro* DC culture system. Our goal was to obtain inflammatory-impaired DCs that would significantly reduce proinflammatory cytokine production upon TLR engagement. Despite the low chemical stability in aqueous buffer, we compared the anti-inflammatory effects of several polyphenols, including capsaicin, resveratrol, retinoic acid, epigallocatechin, curcumin, quercetin and piperine, in *in vitro* cultured DCs. LPS-induced TNFα production was evaluated in treated vs. untreated cells. Quercetin appeared to be consistently the most efficient suppressor of TNFα; these results are consistent with previous reports demonstrating the anti-inflammatory effects of quercetin metabolites[38]. Despite the poor inhibitory properties of TNFα, most of the polyphenols tested were able to suppress IL-6 production. We performed an *in vitro* time course study to evaluate the effects of quercetin. Concomitant administration of LPS and polyphenols appeared ineffective. Administration of polyphenols from the first day of culture induced similar effects to treating twice, on days 3 and 5. TNFα and IL-6 levels detected in the SN of DCs receiving a single dose of polyphenols, either on day 3 or 5, were higher compared to those receiving doses on both day 3 and 5 (data not shown). Since previous reports suggested that piperine acts as a potent bioenhancer for the activity of other polyphenols[39], we evaluated the concomitant administration of both quercetin and piperine. Our results showed that inhibition of both TNFα and IL-6 can be further enhanced when DCs are treated with both quercetin and piperine. Piperine's ability to improve polyphenol inhibition may be due to upregulation of either cytochrome P450, family 4, subfamily f, polypeptide 18 (Cyp4f18) and cytochrome P450, family 4, subfamily a, and/or polypeptide 12a (Cyp4a12a) (data not shown).

Clinical use of polyphenols has been negatively influenced by the chemical characteristics that make them unstable. Indeed, free quercetin is almost completely degraded in 4 hours, and only 30 minutes are needed to observe approximately 50% degradation (Fig. 2F). The mechanism of quercetin degradation in the dark has been proposed to involve the addition of a water molecule to the 2,3 double bond (see structure in fig. 2A) and a further oxidation step, as reported by Dall'Acqua *et al*. (2012)[40]. On the basis of these results, we decided to encapsulate quercetin into ROBs-Q with the intent to improve its stability and bioavailability. As expected, encapsulation of ROBs greatly stabilized quercetin, which showed 100% stability up to three days. These results emphasized the improved efficacy of lipid-based micro/nano particles for quercetin stabilization. We administered ROBs-QP to BMDCs and demonstrated robust suppression of TLR4- and TLR2-mediated inflammatory pathways. The signal cascade involving the P38 pathway was also reduced upon ROBs-QP administration; thus, encouraging further investigation as IBD patients are characterized by macrophages with increased P38α expression. Consistent with other studies demonstrating the ability of LPS and PG to induce COX-2 expression, protein levels of inducible COX-2 were significantly increased in treated cells as early as 30′ with treatment, and remained elevated after 2 hours of stimulation. Interestingly, ROBs-Mix exposed BMDCs, treated with either LPS or PG, significantly reduced p38 MAPK phosphorylation at 10′ and 30′. Single polyphenol ROB administration failed to prevent p38 MAPK phosphorylation. Consequently, in ROBs-Mix exposed BMDCs, COX-2 was significantly reduced 30′ following LPS or PG administration. This effect was lost at 2 h, most likely due to other pathways activated at the same time. We propose that co-treatment with ROBs-P and ROBs-Q might result in a functional synergistic pharmacological effect that goes undetected when only one polyphenol is given, and these effects are at least in part due to the activation of intracellular p38 MAPK.

Following inhibition of the signaling cascade involving the P38 pathway, the release of inflammatory cytokines appeared to be suppressed. Results from dose response studies reveal the pattern of inflammatory cytokine inhibition by ROBs-QP administration. IL-6 is consistently reduced even at a very low dosage (up to 6.25 µM), similarly to other NF-kB-dependent cytokines, such as IL-1α, IL-1β, IL-12, and IL-23. Secretion of anti-inflammatory cytokines, including IL-10 and IL-1Ra, was increased, most efficiently by 25 µM ROBs administration. Nevertheless, it is important to note that the ratio between inflammatory and anti-inflammatory cytokines favors the latter, even at low dosages of ROBs. Production of TGFβ is decreased, as the detectable concentrations of SNs from ROBs-QP treated DCs are similar to untreated DCs. This cytokine secretion path reveals the ROBs-QP DCs become inflammatory impaired. The chemokine profile induced by 25 µM ROBs administration reveals an inhibition of the macrophage inflammatory protein (CCL3), at the same time no significant difference was detected in the production of CCL5 and CXCL1. All together, these data encouraged us to investigate the efficiency of ROBs in an *in vivo* model of acute intestinal inflammation. We evaluated intraperitoneal ROBs-QP treatment starting from day 3 following DSS administration. We decided to start ROB administration when intestinal inflammation is initiated, as our goal was to evaluate a treatment, rather than a prevention, strategy. We evaluated QP presence in the blood and bile of treated mice at 1, 3, 5 and 24 h following ROBs-QP administration. We were able to detect quercetin in the bile of treated mice at 1 and 3 h following ROBs-QP administration although the detected amount was close to the detection limit of the instrument (1 ng). In our experimental conditions, we were never able to detect quercetin in the serum of treated mice. Our results clearly demonstrate that ROBs-QP administration results in a potent reduction of DSS-induced colitis. The small increase in the percentages of MLN CD4^+^Foxp3^+^ T cells may explain the results obtained. Histology from ROBs-QP treated mice indicates a delay in the DSS-induced inflammatory cascade. Furthermore, mice appeared healthy for a longer period, fecal blood was reduced, and survival was prolonged. Colitis is present in both treated and untreated mice exposed to 2% DSS for 9 days, although the histology of ROBs-QP treated mice reveals lower areas of inflammation. Granulocytes infiltrate is comparable in the two groups, consistently with the *in vitro* data showing CXCL1 production unaffected by ROBs-QP administration. The reduced expression of the inflammatory mediator COX2 in the colon lysates with or without epithelial cells indicates that ROBs-QP administration *in vivo* is efficiently affecting lamina propria resident cells including DCs, monocytes and macrophages. Taken together, these results demonstrate that the administration of ROBs containing a polyphenol mix of quercetin and piperine efficiently inhibits LPS-mediated inflammatory responses from DCs. ROB encapsulation protects polyphenols from degradation and improves the efficiency of polyphenol administration. *In vivo*, significant results can be observed in models of acute intestinal inflammation, thus opening new possibilities for novel therapeutic approaches for the treatment of chronic inflammatory diseases.

## Methods

### Ethics statement

C57BL/6 mice were purchased from Charles River Breeding Laboratories. All animal experiments were carried out in accordance with Directive 86/609 EEC enforced by Italian D.L.n 116 1992 and approved by the official RBM veterinarian. When required, euthanasia was performed by cervical dislocation. The protocol was approved by the Committee on the Ethics of Animal Experiments of Ministero della Salute - Direzione Generale Sanità Animale (Prot. 2012/00000923 A00:Eo_GINRC).

### Generation and culture of DCs

DCs were harvested from murine bone marrow (BM). Briefly, BM from the tibiae and femurs of 6- to 8-week-old male C57BL/6 mice were flushed with RPMI and depleted of red blood cells with ACK cell lysing buffer (GIBCO). Cells were plated in 6-well culture plates (1×10^6^ cells/ml; 3 ml/well) in RPMI supplemented with 10% heat-inactivated FBS, 100 U/ml penicillin, 100 mg/ml streptomycin, 25 µg/ml rmGM-CSF, and 25 µg/ml rmIL-4 at 37°C in a humidified 5% CO_2_ atmosphere. On day 3, BMDCs were harvested and plated at 1×10^6^/ml in 24-well culture plates. On day 5 and day 7, BMDCs were administered polyphenols (25 µM). LPS was administered [1 µg/ml] at day 8 for 24 h.

### Enzyme-linked immunosorbent assay (ELISA)

Cell culture SNs were analyzed for TNFα, IL-6, IL-10, IL-12 p70, IL-23, IL-1Rα, IL-1α IL-1β, CCL3 and TGFβ proteins in triplicate, using an ELISA kit, as described by the manufacturer (R&D Systems, Minneapolis, MN).

### Cell cultures and Western blot analysis

BMDCs in primary culture were grown in RPMI 10% FBS in 12 multiwell plates. At day 5 and 7, BMDCs were stimulated with encapsulated polyphenols followed by co-administration of either LPS or peptidoglycan (PG) at various time intervals (10, 30, 120 min) to better evaluate the modulation of intracellular pathway activation downstream of TLR4 or TLR2 agonism. Activation of p42/44 MAPK, p38 MAPK, and NF-κB pathways was assessed. Phosphorylation at Thr^202^/Tyr^204^ and at Thr^180^/Tyr^182^ was considered an indicator of activation for p42/44 and p38 MAPK, respectively. Phosphorylation of IκBα at Ser^32^/^36^, as well as phosphorylation of p65 subunit at Ser^536^, was assumed to be an indicator for NF-κB activation. Cell lysates were prepared using 100 µl of lysis buffer (100 mM NaCl, 40 mM HEPES, pH 7.5, 1% Triton X-100, 1 mM Na_3_VO_4_, 4 mM Na_4_P_2_O_7_, 10 mM EDTA, 1 mM PMSF, 10 mM NaF, 2 µg/ml aprotinin, and 2 µg/ml leupeptin). Equal amounts of protein (25 µg) were separated by 10% SDS-PAGE and subjected to immunoblotting with the following primary antibodies (dilution 1∶1,000): COX-2, p65, ph-p65, (Santa Cruz Biotecnology, Santa Cruz, CA); IκBα, ph-IkBα, ERK1/2, ph-ERK1/2, p38 MAPK, and ph-p38 MAPK, (Cell Signaling Technology). The β-actin antibody was from Sigma. Incubation with horseradish peroxidase-linked anti-mouse, anti-rabbit, and anti-goat secondary antibodies (Santa Cruz Biotecnology) (1∶3,000) was performed for 1 h at room temperature. Immunoblotting results were visualized by Molecular Imager ChemiDoc XRS System (Bio-Rad Laboratories). Images were captured with QuantityOne Software (Bio-Rad Laboratories) and blots quantified by scanning densitometry (Image J; National Institutes of Health, Bethesda, MD).

### Histological examination

Tissue section from the distal colon were fixed in 10% buffered formalin and embedded in paraffin. Sections of 6 µm were stained with hematoxylin and eosin (H&E). Images were acquired using Leica LMD 6500.

### Purification of OBs

OBs were extracted from almond seeds. OB purification was carried out by a two-layer flotation procedure as previously reported [22], and a further purification step was performed consisting of two sequential washings with 2.0 M NaCl. OBs were finally resuspended in 150 mM Tris–HCl, pH 7.5, containing 0.6 M sucrose.

### Quercetin and piperine encapsulation into ROBs

Quercetin and piperine were encapsulated into the ROBs using the above reported protocol [22] with few modifications. Natural OBs were resuspended in 150 mM TRIS-HCl pH 7.5, containing 0.5 M sucrose, 1 mM EDTA, 10 mM KCl, 1 mM MgCl_2_, 5 mM ascorbic acid (buffer A) and twice extracted with chloroform: methanol (2∶1), in order to separate PLs and proteins from TAGs. After centrifugation at 1000 *g* for 5 minutes, the upper phase was extracted with five volumes of diethyl ether anhydrous (to recover TAGs). All the recovered fractions were dried by a rotavapor.

ROB reconstitution mixture consisted of the whole chloroform-methanol phase (PLs) with a quercetin (or piperine)/TAGs ratio of 1/250 starting from 2 mg of each phytochemical. The final volume was adjusted to 3 ml by the addition of buffer A. Samples were sonicated by a Brandson digital Sonifier 250-D at an amplitude of 40% and a cycle of 30 seconds pulse on and 30 seconds off for three times. After sonication, samples were centrifuged at 2000 *g* for 10 minutes and ROBs containing quercetin or piperine were recovered from the top of the centrifuge-tubes.

HPLC analysis was carried out extracting the encapsulated phytochemicals using 2 volumes of ultrapure water, 2 volumes of methyl-butyl-ether, and 1 volume of methanol. After centrifugation at 1000 *g* for 5 minutes, the methyl-butyl-ether phase was recovered, dried (rotavapor), and resuspended into 50% ethanol. Quercetin was quantified by reverse phase-HPLC using the method described above [41] onto a 1100 Agilent work chromatographic station equipped with a photodiodes array UV-Vis detection system. The elution profile was monitored at 370 nm. Quercetin, purchased from Sigma-Aldrich, was used as standard.

Quercetin-loaded ROBs were imaged by confocal microscopy using an excitation wavelength of 488 nm; emission was recorded with a 505–530 nm filter set. Empty ROBs were imaged by transmitted light microscopy. Confocal micrographs were taken with a Leica confocal scanning system mounted to a Leica TCS SP5 (Leica Microsystem GmbH, Mannheim, Germany).

### *In vivo* treatment with nanocapsules

Acute colitis was induced by administration of 2% DSS in drinking water. Sex- and weight-matched mice were divided in 2 groups (10 mice each). 2% DSS in drinking water was administered starting from day 0. Starting at day 3, mice were either injected intraperitoneally with ROBs-QP [0.5 µM/g] or vehicle three times per week. Mice were monitored on a daily basis for the following 10 days. Body weight, stool consistency, and rectal bleeding were recorded. Mice were sacrificed at day 10, and colon and MLN tissues were explanted to evaluate clinical severity of colitis. Colon length was measured as an indicator of colonic inflammation. Colons were placed onto a non-absorbent surface and measured with a ruler, taking care not to stretch the tissue. The colon/body weight index was calculated as the ratio of the colon wet weight and the total body weight (BW) of each mouse. Body weight, occult and rectal bleeding, and stool consistency were monitored daily after DSS administration. Disease activity index (DAI) was determined by scoring change in body weight, occult blood and gross bleeding as described in the literature[42] [43]. Single cell preparations obtained from the MLNs were used to evaluate CD4^+^Foxp3^+^ cells according to the flow cytometry FOXP3 staining kit (eBioscience). Epithelial cells layer was mechanically stripped from the colon. The colon was cut in half, longitudinally opened and cleaned. The colon sections were washed 3 times with 2.5 mM EDTA at 4°C, the epithelial cells containing supernatant removed and the colon lysated.
